# Transient PP2A inhibition alleviates normal tissue stem cell susceptibility to cell death during radiotherapy

**DOI:** 10.1038/s41419-018-0559-0

**Published:** 2018-04-30

**Authors:** Maria Rita Fabbrizi, Barbara Meyer, Sandeep Misri, Suyash Raj, Cheri L. Zobel, Dennis E. Hallahan, Girdhar G. Sharma

**Affiliations:** 1 Cancer Biology Division, Department of Radiation Oncology, Washington University School of Medicine, 4511 Forest Park, Saint Louis, MO 63108 USA; 2 Siteman Cancer Center, Washington University School of Medicine, Saint Louis, MO 63108 USA

## Abstract

Unintended outcomes of cancer therapy include ionizing radiation (IR)-induced stem cell depletion, diminished regenerative capacity, and accelerated aging. Stem cells exhibit attenuated DNA damage response (DDR) and are hypersensitive to IR, as compared to differentiated non-stem cells. We performed genomic discovery research to compare stem cells to differentiated cells, which revealed Phosphoprotein phosphatase 2A (PP2A) as a potential contributor to susceptibility in stem cells. PP2A dephosphorylates pATM, γH2AX, pAkt etc. and is believed to play dual role in regulating DDR and apoptosis. Although studied widely in cancer cells, the role of PP2A in normal stem cell radiosensitivity is unknown. Here we demonstrate that constitutively high expression and radiation induction of PP2A in stem cells plays a role in promoting susceptibility to irradiation. Transient inhibition of PP2A markedly restores DNA repair, inhibits apoptosis, and enhances survival of stem cells, without affecting differentiated non-stem and cancer cells. PP2Ai-mediated stem cell radioprotection was demonstrated in murine embryonic, adult neural, intestinal, and hematopoietic stem cells.

## Introduction

Ionizing radiation (IR) is a major cancer treatment modality for primary and metastatic cancers, but invariably results in debilitating organ dysfunction such as cognitive impairment^1,2^ and learning deficiencies in patients subjected to cranial irradiation^3,4^. Similarly, IR therapy-induced intestinal injury is a common problem in patients with abdominal and pelvic cancers and is associated with a loss of stem cells^5^. IR response of progenitor cells is determined mostly by the intrinsic radiation hypersensitivity and unique molecular/epigenetic regulation of DNA damage response (DDR) and apoptotic response (AR) in stem cells^6–8^. Although all the mechanistic regulation of stem cell radiosensitivity has not been elucidated, the differential expression of several genes in stem cells plays a role in attenuated DDR and heightened AR^6^. For example, histone modifications that are unique to stem cells include Histone 3 Lysine 56 acetylation (H3K56ac)^7^ and H3K9 acetylation/methylation^8^.

Embryonic stem (ES) cells in culture maintain the stem cell phenotype and provide a discovery tool when compared to differentiated (ED) cells. We compared the gene expression of ES and ED cells and found that Phosphoprotein Phosphatase 2A (PP2A) contributes to DDR signaling and is associated with the radiosensitivity observed in normal stem cells. PP2A activity has also been associated with maintenance of “stemness”^9^. PP2A holoenzyme participates in many cellular functions such as neural growth, replication, and several metabolic pathways^10,11^. PP2A dephosphorylates pATM and γH2AX, and deactivates DDR once the DNA strand break (DSB) is repaired^12^. In addition, PP2A dephosphorylates Akt at both Thr308 and Ser473 sites, resulting in consequent apoptotic pathway activation^13^, and PP2A inhibition has been suggested as potential cancer treatment and knockdown of PP2A in several in vitro cancer cell models resulted in elevated γH2AX and increased radiosensitivity^14–17^. However, recent studies suggest PP2A activation as potential tumor suppressor and indicate promising results in chemotherapeutic treatment of cancers^18^, therefore further studies are needed to elucidate the mechanisms. The role of PP2A in stem cell response during the DDR was studied in the experiments presented herein. We hypothesized that PP2A phosphatase antagonizes DNA repair and is a unique molecular switch that imparts differential response to DNA damage in stem cells. We compared karyotypically normal, early passage, radiosensitive stem cells with isogenic, differentiated cells to delineate the role of PP2A during the DNA damage and apoptotic responses.

We thus show that PP2A contributes to stem cell radiosensitivity in murine intestinal organoids, neural, and hematopoietic stem cells all of which belong to the tissues that demonstrate high radiosensitivity in their stem cell compartment. Transient suppression of PP2A significantly decreased stem cell radiosensitivity, reduced IR-induced apoptosis, and improved stem cell survival without affecting differentiated cells or cancer cells. In addition, we observed PP2Ai-mediated reduction in IR-sensitivity in human neuroprogenitor cells. PP2A inhibition may be a therapeutic approach for radioprotection of normal tissue stem cells during radiotherapy in cancer patients.

## RESULTS

### PP2A is constitutively overexpressed in stem cells in vivo and in culture

**Fig. 1:**
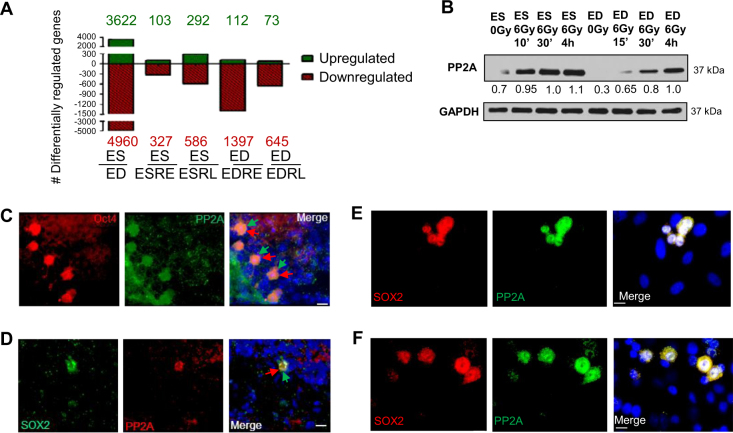
PP2A is constitutively overexpressed in stem cells in vivo and in culture. **a** The number of genes up- or downregulated more than two-fold is compared between ES and ED cells as well as among irradiated ES and ED cells collected early (15 min; RE, radiation early) or late (4 h; RL, radiation late) after 10 Gy IR using microarray analysis. **b** PP2A and GAPDH were detected in lysates of ES and ED cells using immunoblot. **c** Tissue sections obtained from testis of WT C57BL/6 mice were stained with Oct4, PP2A, and DNA labeled with DAPI. **d** Tissue sections of the dentate gyrus of hippocampus obtained from brains of WT C57BL/6 mice were stained with SOX2, PP2A, and DNA labeled with DAPI. **e** ES and ED were co-plated and stained with SOX2, PP2A, and DNA labeled with DAPI. Cells were stained after 0 Gy treatment. **f** ES and ED were co-plated and stained with SOX2, PP2A, and DNA labeled with DAPI. Cells were stained 30 min after 6 Gy treatment. Scale bars indicate 10 μm

To identify the unique regulatory mechanisms underlying stem cell radiation response, gene expression profiles of isogenic ES and ED cells^7,8^ were compared before and after radiation treatment using genechip microarray analysis. With the aim of finding contrasting gene expression patterns, differential alterations in expression profiles were investigated at an early time point of 15 min (radiation early, RE) and at a late time point of 4 h (radiation late, RL) after irradiation on ES and ED cells. Differentiation of stem cells led to transcriptional induction of 3622 genes, whereas 4960 genes were suppressed (Fig. 1a). Of these 8582 genes, expression of 139 genes was commonly altered when stem cells underwent differentiation, as well as after irradiation of differentiated cells. In contrast, expression of 144 genes was commonly altered following both differentiation and irradiation in stem cells (Supplemental Fig. S1A). We reason that these unique subsets of genes in the intersection lists (Supplemental Table 1, the raw data of gene-expression profiling at NIH’s Gene Expression Omnibus can be found at http://www.ncbi.nlm.nih.gov/geo/query/acc.cgi?token=hpexbaiyaoawyre&acc=GSE44780) contain potential molecular regulators of the radiosensitive and radioresistant phenotypes of stem and non-stem cells, respectively. Pathway analysis, enrichment scores, and functional interaction determinations using GeneGo analyses showed that the most pronounced IR-induced change in the stem cell transcriptome was associated with affecting biosynthesis such as ribosome expression. The non-stem cell transcriptome, however, promoted activation of survival signaling pathways as well as protein digestion and absorption (Supplemental Fig. S1B). Through rigorous statistical short listing and verification analyses, we identified overexpression and substantial radiation induction of the catalytic subunit of phosphoprotein phosphatase 2A (PP2A) selectively in stem cells, which was confirmed by RT-PCR (Supplemental Fig. S1C).

Immunoblotting confirmed that ES cells express detectable basal levels of PP2A without IR treatment. After 6Gy irradiation, PP2A expression increased dramatically at 10 min to 4 h post IR treatment, while in ED cells PP2A increased gradually and reached the maximum expression after 4 h (Fig. 1b), when it is presumed to dephosphorylate pATM and γH2AX toward the end of DSB repair^12,19^.

We treated cells with 0.8 nM Calyculin A (Cal A), a known chemical inhibitor of PP2A phosphatase. We measured the phosphatase activity by specific immunoprecipitation kit (PP2A Immunoprecipitation Phosphatase kit, Milllipore) and observed a clear reduction of PP2A activity in stem cells (Supplemental Fig. S1D). We also genetically silenced PP2A using a pool of several specific siRNAs and observed a reduction of PP2A protein levels after 36 and 48 h of RNAi treatment (Supplemental Fig. S1E).

To validate whether PP2A is overexpressed in stem cells in vivo, we studied stem cell niches in the brain, intestine and testis. Identification of adult spermatogonial stem cells in murine testis has been obtained with PLZF-positive^8^ and Oct4-positive staining. Stem cells showed significantly higher levels of PP2A compared to the surrounding non-stem cells (Fig. 1c). Similar overexpression was also detected in neural stem cells (Fig. 1d) and in intestinal stem cells (Supplemental Fig. S1F). Immunofluorescent staining on co-plated ES and ED culture cells further confirmed PP2A overexpression in ES cells (Fig. 1e, f). Almost 85% of cells that stained positively for stem cell markers showed overexpression of PP2A, both in vivo and in culture, compared to only 5% of differentiated cells which were negative for stem cells marker. PP2A was differentially overexpressed in murine embryonic stem cells, neuro-progenitors, intestinal, and spermatogonial stem cells in comparison to their non-stem progeny cells.

### Suppression of PP2A restores DNA repair in stem cells

**Fig. 2:**
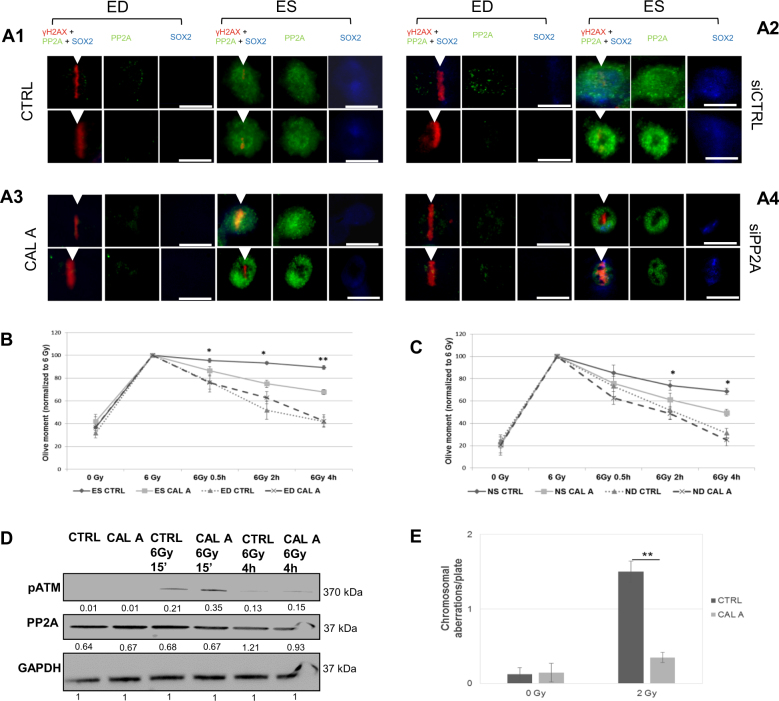
Suppression of PP2A restores DNA repair in stem cells. ES and ED cells were treated with Calyculin A (Cal A), control siRNA (siCtrl), or PP2A siRNA (siPP2A) or left untreated. Scale bars indicate 10 μm. Error bars indicate SD; *p < 0.05; **p < 0.01. Three independent experiments were performed. **a** Cells shown in all panels were microirradiated, fixed immediately after irradiation and stained for PP2A and γH2AX detected along with Sox2 (arrow indicates laser region of interest, ROI). (a1) Untreated ED (left panel) and ES cells (right panel). (a2) ED (left panel) and ES cells (right panel) treated with siCtrl. (a3) ED (left panel) and ES cells (right panel) treated with Calyculin A. (a4) ED (left panel) and ES cells (right panel) treated with siPP2A. **b** ES and ED cells treated with Calyculin A or untreated were analyzed by comet assay. Values were normalized to 6 Gy time point. **c** NS and ND cells treated with Calyculin A or untreated were analyzed by comet assay. Values were normalized to 6 Gy time point. **d** ATM-pS1981 (pATM), PP2A, and GAPDH were detected in lysates of ES cells using immunoblot. **e** Chromosomal aberrations per set of chromosomes were quantified at 7 h after 2 Gy irradiation

We co-plated ES and ED cells and microirradiated ES cells in close proximity to ED cells. The induction of DDR on the DSBs along the micro-irradiation track was visualized by labeling γH2AX in ES and ED cells while ES cells were identified by SOX2 positive staining (Fig. 2a). Eighty percent of the untreated ES cells and ES cells treated with CTRL siRNA showed high pan-nuclear PP2A levels (Fig. 2a1, a2), while displaying a significantly reduced γH2AX signal at DNA lesions compared to ED cells (Supplemental Fig. S2(A)). In contrast, ES treated with PP2A inhibitors (chemical inhibitor Cal A (Fig. 2a3) and siPP2A pool treated (Fig. 2a4)) showed a clear γH2AX signal at the DNA break site, with a decrease in PP2A signal intensity in siRNA-treated cells (Supplemental Fig. S2(A)). The non-stem cells showed mostly undetectable or reduced PP2A levels and a clear γH2AX activation in all samples, indicating that PP2A inhibition did not exert any effect on differentiated cells (Supplemental Figs. S2(A1–4)). Detection of γH2AX with flow cytometry corroborated improved DDR activation after PP2A inhibition (Supplemental Fig. S2(B)A).These results indicate differential regulation and dissimilar involvement of PP2A in DDR in stem vs. non-stem cells.

We measured the DSB repair efficiency and kinetics by neutral comet assay after PP2A inhibition/suppression in ES and neural stem cells (NS). An improved DNA DSB repair efficiency was observed in ES cells after treatment with Cal A (Fig. 2b) and after RNAi suppression (Supplemental Fig. 2(B)B), while inhibition/suppression in the non-stem ED cells showed no influence on DNA repair. The same result was observed in NS cells with no effect on non-stem differentiated ND cells (Fig. 2c and Supplemental Fig. 2(B)C).

We next investigated the effect of PP2A inhibition upon ATM activation in stem cells and observed a significant increase in ATM activation 15 min post IR in ES cells treated with Cal A compared with untreated samples (Fig. 2d). Cytogenetic analysis also confirmed an improved chromosomal repair and reduced residual chromosome and chromatid breaks in ES that have been treated with PP2A inhibitor (Fig. 2e).

### PP2A inhibition suppresses apoptosis signaling in stem cells

**Fig. 3:**
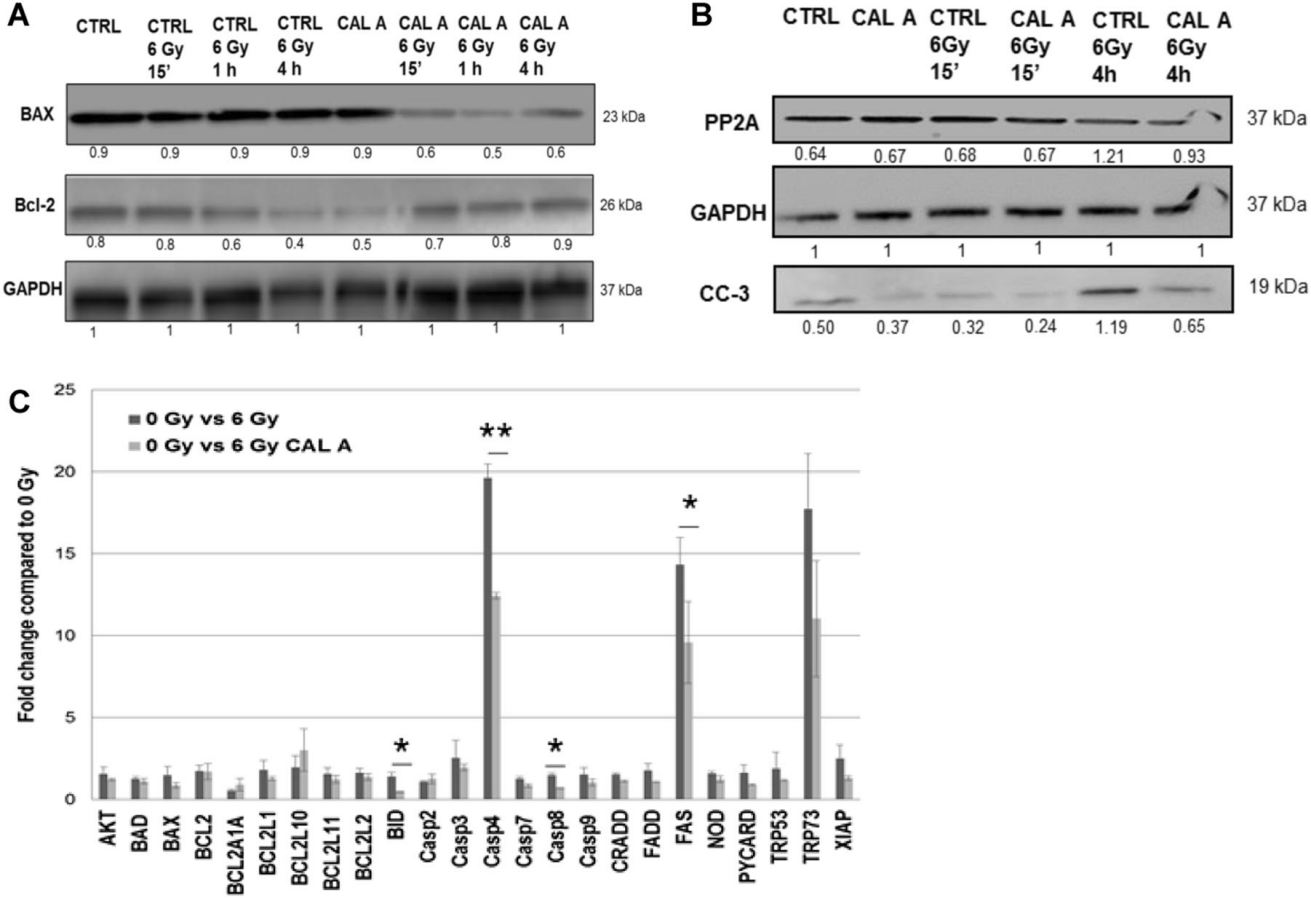
PP2A suppression influences apoptosis signaling. ES cells were treated with Calyculin A (Cal A) or left untreated. Error bars indicate SD; *p < 0.05; **p < 0.01. **a** Bax, Bcl-2, and GAPDH were detected in lysates of ES cells using immunoblot. **b** PP2A, GAPDH, and CC-3 were detected in lysates of ES using immunoblot. **c** Apoptotic gene expression was quantified using qRT-PCR in ES cells 4 h after 6 Gy IR or without IR. The data were normalized to the expression levels in untreated ES cells. Three independent experiments were performed

To investigate whether PP2A inhibition has direct or indirect influence on the apoptotic signaling pathways in irradiated stem cells, we performed transient knock down of PP2A and analyzed pro- and anti-apoptotic proteins by immunoblotting. PP2A chemical inhibition resulted in reduced Bax, which has been found to be constitutively elevated in stem cells^20^, increased Bcl-2 protein levels compared to ES control (Fig. 3a) and led to a marked reduction of cleaved caspase-3 levels (Fig. 3b). We also observed an increased activation of Akt after Cal A treatment, which is normally absent in ES cells after IR (Supplemental Fig. S3A). To obtain a more holistic view of PP2Ai effects on apoptotic network proteins, we performed RT-PCR analysis on a panel of apoptosis regulatory genes (RT² Profiler™ PCR Array Mouse Apoptosis from Qiagen). This analysis showed a clear reduction in expression of several genes involved in the apoptotic pathway in ES treated with Cal A compared to the 6 Gy control, with a dramatic decrease in caspase-4 and Fas expression (Fig. 3c).

### Influence of PP2Ai is mediated by both ATM and Akt

**Fig. 4:**
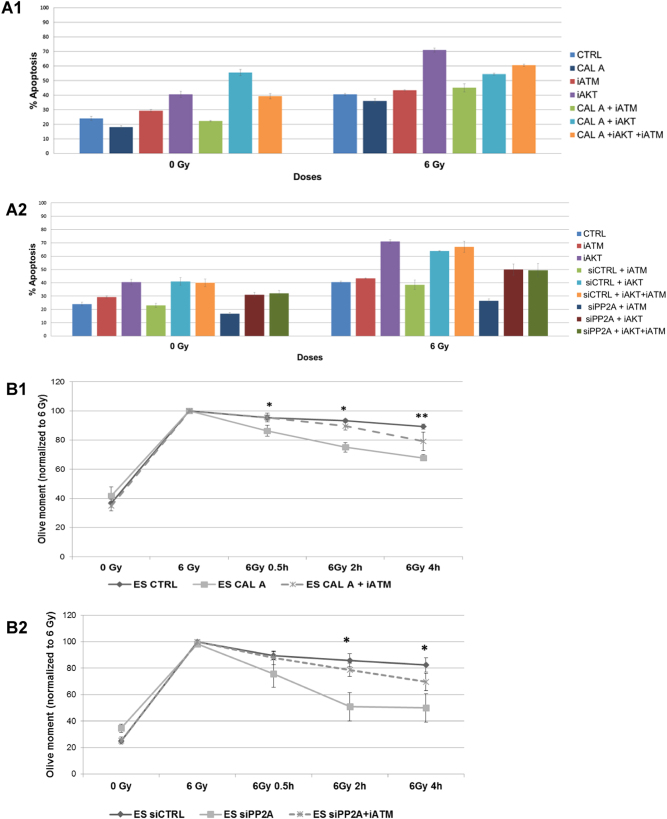
Influence of PP2Ai is mediated by both ATM and Akt. ES cells were treated with Calyculin A, control siRNA (siCtrl) or PP2A siRNA (siPP2A) or left untreated. Error bars indicate SD; *p < 0.05; **p < 0.01. Three independent experiments were performed. **a1** ES cells were either untreated controls or they were treated with Calyculin A, together with ATM and/or Akt inhibitors. Cells were irradiated with 6 Gy and apoptosis analyzed after 16 h by Annexin V labeling. **a2** ES cells were treated with control siRNA (siCtrl) or PP2A siRNA (siPP2A), together with ATM and/or Akt inhibitors. Cells were irradiated with 6 Gy and apoptosis analyzed after 16 h by Annexin V labeling. **b1** ES cells were untreated or treated with Calyculin A, together with ATM inhibitor analyzed by comet assay. Values were normalized to 6 Gy time point. **b2** ES cells were treated with siCtrl or PP2A siRNA with ATM inhibitor analyzed by comet assay. Values were normalized to 6 Gy time point

PP2A is known to have a role in the deactivation of DDR by dephosphorylation of pATM and γH2AX^12,19^. PP2A also dephosphorylates Akt at both Thr308 and Ser473 sites, with consequent apoptotic pathway activation^13^. We analyzed PP2Ai influence on ATM and Akt by Annexin V and neutral comet assay and observed that simultaneous inhibition of PP2A and ATM/Akt withdrew the radioprotective effect of PP2Ai on ES cells. However, a detrimental effect of Akti was observed with high levels of apoptosis (Fig. 4a1, a2, statistical analysis in Supplemental Fig. 4A). DNA repair efficacies were also reduced drastically after simultaneous inhibition of PP2A and ATM (Fig. 4b1, b2).

### Suppression of PP2A reduces IR sensitivity of stem cells

**Fig. 5:**
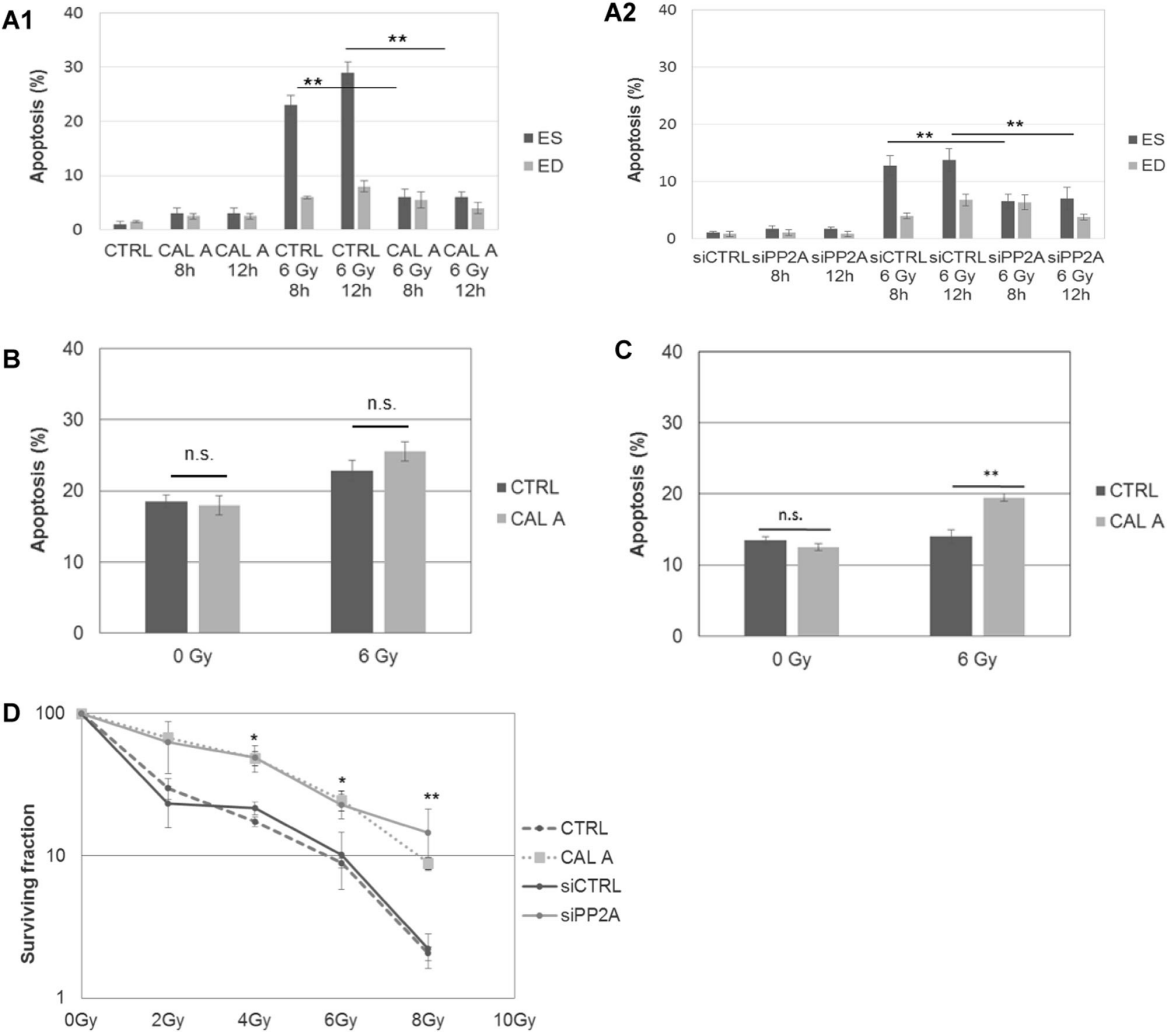
Suppression of PP2A reduces IR sensitivity of stem cells. ES and ED cells were treated with Calyculin A (Cal A), control siRNA (siCtrl), or PP2A siRNA (siPP2A) or left untreated. Error bars indicate SD; *p < 0.05; **p < 0.01; n.s. = not significant. Three independent experiments were performed. **a1** ES and ED cells were treated with Cal A, irradiated with 6 Gy and apoptosis analyzed after 8 and 12 h by Annexin V labeling. **a2** ES and ED cells were treated with siPP2A, irradiated with 6 Gy and apoptosis analyzed after 8 and 12 h by Annexin V labeling. **b** Human medulloblastoma cells (Daoy HTB-186) were treated with Cal A or left untreated. Cells were irradiated with 6 Gy and apoptosis analyzed after 16 h by Annexin V labeling. **c** Murine glioblastoma cells (GL261) were treated with Cal A or left untreated. Cells were irradiated with 6 Gy and apoptosis analyzed after 16 h by Annexin V labeling. **d** Clonogenic survival of ES cells was analyzed after the indicated doses of irradiation

After observing the improved DNA repair and reduced apoptotic signaling, we then investigated whether the inhibition of PP2A was effective at radioprotecting stem cells and improving cell survival. Flow cytometric analysis of Annexin V revealed a remarkably significant decrease in apoptosis selectively in irradiated ES cells with depletion of PP2A activity by chemical inhibition (Fig. 5a1) and expression by RNAi (Fig. 5a2), while no protective effect was observed in medulloblastoma (Fig. 5b and Supplemental Fig. 5A) and glioblastoma cancer cells (Fig. 5c and Supplemental Fig. 5B). Clonogenic assay reasserted the decreased radiosensitivity and the improved clonogenic survival of irradiated ES cells after PP2A downregulation (Fig. 5d).

### PP2A inhibition decreases radiosensitivity of stem cells in ex vivo organoid cultures

**Fig. 6:**
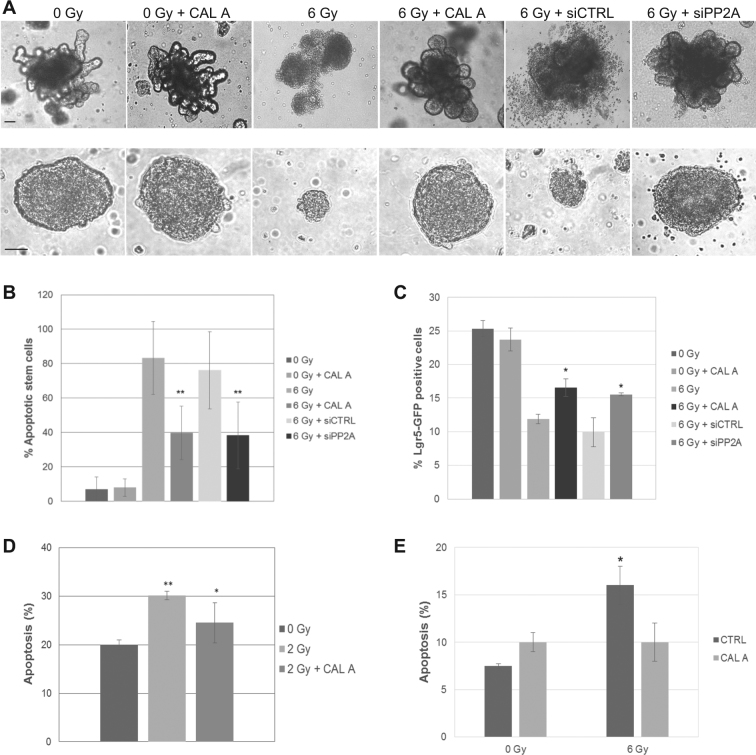
PP2A inhibition decreases radiosensitivity of stem cells in ex vivo organoid cultures. **a** Shown are optical microscope images of murine intestinal organoids (upper panel) and murine neurospheres (lower panel) untreated or irradiated at 6 Gy after treatment with Calyculin A (Cal A), control siRNA (siCtrl), or PP2A siRNA (siPP2A). Pictures have been taken 48 h after IR treatment. **b** Murine intestinal organoid stem cells were left untreated or irradiated at 6 Gy after treatment with Calyculin A (Cal A), control siRNA (siCtrl) or PP2A siRNA (siPP2A). Percentage of cells positive for stem cell marker SSEA1 and apoptotic marker CC-3 was calculated from total SSEA1 positive cells. Scoring was performed 4 h after irradiation. **c** Murine intestinal organoid stem cells were left untreated or irradiated at 6 Gy after treatment with Calyculin A (Cal A), control siRNA (siCtrl), or PP2A siRNA (siPP2A). Percentage of cells Lgr5-GFP positive was detected by flow cytometry. Scoring was performed 4 h after irradiation. **d** Murine hematopoietic stem cells were treated with Cal A or left untreated. Cells were irradiated with 2 Gy and apoptosis analyzed after 16 h by Annexin V labeling. **e** Human neuroprogenitors were treated with Cal A or left untreated. Cells were irradiated with 6 Gy and apoptosis analyzed after 16 h by Annexin V labeling. Scale bar = 100 μm. Error bars indicate SD; *p < 0.05; **p < 0.01. Three independent experiments were performed

We have so far established that PP2A plays dual regulatory role in DDR and AR responses in stem cells in culture models. We employed the 3D ex vivo organoid explant culture models to verify the radioprotective efficacy of PP2A. PP2A inhibition led to better preservation of 3D structures, maintenance, and growth of both murine intestinal organoids grown on matrigel and neurospheres grown in suspension cultures (Fig. 6a). Immunofluorescent identification of crypt stem cells in the intestinal organoids by staining with intestinal stem cell marker SSEA1 and co-labeling with CC-3 revealed selectively high apoptosis in stem cells after IR in the organoid model and a clear reduction after PP2A inhibition (Fig. 6b). Proliferative cells marked with Ki67 positivity largely comprised SSEA1-negative non-stem cells in the organoids and did not show any significant improvement in cell survival after IR treatment (Supplemental Fig. 6A). To further confirm the immunostaining based observations, we utilized the Lgr5-EGFP-IRES-creERT2 mouse model, where intestinal stem cells are GFP positive^21,22^. Cells dissociated from organoids grown from the small intestine of a Lgr5-EGFP-IRES-creERT2 mouse were analyzed by flow cytometry for GFP positivity with or without PP2Ai after 6 Gy IR treatment. The number of Lgr5-GFP-positive cells increased after Cal A or PP2A RNAi treatments compared to the cells from 6 Gy control organoid samples from the Lgr5-GFP mouse (Fig. 6c). PP2A knockdown efficiency in intestinal organoids using siRNA pool was confirmed by reduced PP2A levels on immunoblots (Supplemental Fig. 6B). We also observed PP2Ai mediated reduction of IR-induced apoptosis in the murine hematopoietic stem cells (Fig. 6d). Besides all the aforementioned murine embryonic and adult stem cell models, we further ascertained the radioprotective effect of transient PP2Ai in the human neuroprogenitors (Fig. 6e). Collectively, these data provide a mechanistic basis and corroboratively reinforce the potential therapeutic efficacy of PP2Ai in prevention of radiotherapy induced stem cell death.

## DISCUSSION

Radiotherapy-induced stem cell depletion is believed to impair regenerative abilities of many tissues. Research findings in recent years suggest that stem cell transplant in addition to the use of anti-inflammatory agents may provide a useful intervention strategy for minimizing the adverse effects of cranial irradiation on central nervous system (CNS) function. However, application of stem cell transplant therapy to alleviate cognitive deficits in CNS malignancy treatment regimen has enormous practical limitations. Normal stem cells in multiple tissue niches have been shown to be deficient in DNA repair and undergo IR-induced programmed cell death even at low doses that do not kill non stem and cancer cells. We envisage that normal stem cell radiosensitivity is a phenomenon regulated by pluralistic factors that attenuate DDR and promote AR. Our laboratory has identified and characterized a few epigenetic “structural” barriers of DDR in stem cells previously^7,8^. Here we have found constitutively overexpressed and IR-induced PP2A phosphatase as a “signaling” barrier that promotes radiosensitivity in normal stem cells. However, besides PP2A and histone modification profiles characteristic of stem cells, other regulators may also have important roles in imparting the radiosensitive phenotype (Supplementary Table 1). These molecular regulators collectively diminish DDR and DNA repair and promote AR in stem cells in a multifaceted manner.

Here we propose a pivotal regulatory role of PP2A phosphatase in promoting normal stem cell radiosensitivity. Although PP2A role has been investigated in differentiated and cancer cells and its inhibition has been proven to cause delay in tumor growth, the significance of PP2A overexpression in stem cells remains unknown. PP2A phosphatase is an antagonist of ATM/DDR signaling that inhibits DNA DSB repair as well as promotes apoptosis. Transient suppression of PP2A activity or expression markedly improves ATM activation, restores DNA repair, inhibits apoptosis and enhances survival of stem cells without any significant effect on differentiated non-stem cells and cancer cells. Prolonged inhibition or knockdown of PP2A led to cytotoxicity and cytostatic effects. PP2A is known to be involved in several cell processes including cell proliferation, growth, and survival^10,11^. It also dephosphorylates pATM and γH2AX and is believed to be a turn off switch for DNA repair machinery at the end of DSB repair^12,19^. PP2A inhibition has been suggested as potential cancer treatment and knockdown of PP2A in several in vitro cancer cell models resulted in elevated γH2AX and increased radiosensitivity^14–17^. Our observation on glioma cells GL261 showed a similar trend. However, some studies have also indicated that PP2A suppression reduces apoptosis in some cancer cell lines^23,24^ and the concept of activating PP2A as potential tumor suppressor has indicated positive results in chemotherapeutic treatment of several cancers^18^. Despite extensive studies on the role of PP2A in different cellular processes in various cancer cell lines, little is known about the radioprotective effect of PP2A suppression in stem cells. Li and colleagues observed that suppression of PP2A subunit B56ϵ in human embryonic kidney epithelial cell line caused γH2AX to persist on the DSB site, leading to increased chromatin instability, diminished cell cycle regulation, and DNA repair^25^. Our results obtained from normal stem cell models clearly demonstrate that elevated PP2A levels in stem cells prohibit ATM activation after IR treatment and that transient suppression of PP2A successfully restores the activity of ATM at the DNA breaks and improves DSB repair. We also observed that histone H2AX S139 phosphorylation around DNA breaks is attenuated in stem cells and PP2A suppression restores γH2AX at DNA breaks, confirming that reduction of PP2A activity induces DDR activation in normal stem cells, as observed in other non-stem cell types^12^. Furthermore, simultaneous inhibition of PP2A and ATM partially obliterates the restoration of DDR and DNA repair in stem cells, indicating that the radioprotective action of PP2A is predominantly ATM dependent. PP2A also dephosphorylates the serine/threonine protein kinase B (PKB, also known as Akt) which plays an essential role in several cellular processes such as survival, growth, proliferation^26,27^, self-renewal of stem cells^28^ and has also been associated with cancer and neurodegenerative diseases when mutated^29–31^. Cancer cells exposed to PP2A inhibitor show elevated Akt phosphorylation and activation^32–34^. In agreement with these findings, we found that PP2A inhibition increases Akt phosphorylation in normal stem cells and that simultaneous inhibition of PP2A and Akt increases apoptosis compared to cells treated with only Cal A or siPP2A. Slight increase in the apoptosis in glioma cells by PP2Ai is likely due to the activation of Akt and suggests the anti-cancer prospects of PP2A inhibition^35^.

PP2A regulates apoptosis by dephosphorylating anti-apoptotic factor Bcl-2 and by activating pro-apoptotic Bad and Bax^36,37^. PP2A also regulates p53 signaling through ATM and Akt^38,39^ and by direct dephosphorylation of p53, promoting cell cycle arrest, expression of Bax and apoptosis^40,41^. The precise mechanistic role(s) of PP2A activity in apoptosis pathways is still not entirely clear. PP2A inhibition has been found to drastically reduce apoptosis in myeloid cells^42,43^ but not in other cancer cell lines^44^ or in endothelial cells^45^. In contrast, treatment of cancer cells with potent PP2A activators or overexpression of the PP2A catalytic subunit inhibits Bcl-2 phosphorylation, leading to increased p53/Bcl-2 binding and apoptotic cell death^9,42^. We observed that in stem cells inhibition/RNAi of PP2A leads to suppression of the apoptotic pathway with a significant decrease of apoptotic cells, lowered levels of pro-apoptotic Bax protein and elevated levels of anti-apoptotic Bcl-2. Besides, we also observed significantly elevated expression of several regulators involved in programmed cell death pathways such as Fas, CRADD, and caspase-4, probably caused by the upregulation of endoplasmic reticulum stress marker noticed after IR treatment^46–48^. Cal A treatment increases anti-apoptotic Bcl-2 protein level and dramatically decreases the expression of several caspases. While DDR is resumed primarily through ATM activation, the concurrent effects on reduced apoptosis is multifactorial. PP2A-caspase-4 interaction and other interacting mechanisms underlying PP2Ai-mediated radioprotection need further investigation. Our data highlights the multifaceted role of PP2A in regulation of stem cell radiosensitivity and the functional dichotomy of PP2A in normal stem cells vs. cancer cells makes PP2A an interesting molecular target for radioprotecting stem cells.

Although we have established radioprotection in cellular models of stem cells, toxicity of 0.8 nM Cal A (at which it specifically inhibits PP2A phosphatase activity) exposure for more than 2–3 h in culture and toxicity of Cal A to mice prohibited us from validating the PP2Ai radioprotective efficacy in tissue niches in vivo. We therefore utilized the ex vivo three-dimensional (3D) organoid explant models in the present study. We successfully grew murine intestinal organoids from stem cells from wild type mice and from genetically modified mice which present Lgr-5-positive intestinal stem cells labeled with GFP. IR treatment resulted in loss of 3D structure of the organoids along with an increased number of apoptotic stem cells in both the organoid models. Differentiated, actively proliferating non-stem cells in the intestinal organoids show no benefit from treatment with Cal A or siPP2A and appeared to be more radioresistant implicating the differential regulation of radiosensitivity in stem vs. non-stem cells. The loss of 3D structure has been observed before in ex vivo crypts after increasing irradiation doses^49^ and in vivo data show that 10% of intestinal stem cells initiate apoptosis after low doses, without any appreciable alteration in the intestine architecture^7,50,51^. While organoids do resemble the physiology and cell architecture of the intestine, they manifest eventual disruption of 3D structure unlike the resilient intestine in vivo, possibly due to lack of some essential factors and supportive tissue that is present in vivo but not in the explant organoid cultures in matrigel^52^. PP2A inhibition successfully improved cell survival in intestinal organoids, which showed a reduced number of SSEA1/CC-3 double positive cells, and helped in maintaining the 3D structure of the ex vivo organoid cultures.

Thus, using cellular, ex vivo organoids and in vivo models, this study has addressed the associations between cellular differentiation, DNA damage response and apoptotic response unique to stem cells and characterized multifaceted role of PP2A in antagonizing DDR and promoting AR. Corroborative validation of transient PP2A suppression in radioprotection of stem cells emphasizes PP2A as a novel molecular target for radioprotection. This will allow future development of innovative prevention and intervention strategies to alleviate the undesired side effects of radiotherapy which impairs the quality of life of cancer survivors especially in pediatric neoplasms.

## MATERIALS AND METHODS

### Cell culture and undirected differentiation

Wild-type murine embryonic stem cells were originally isolated from mouse blastocysts and obtained from the Murine Embryonic Stem Cell Core at Washington University in Saint Louis. Cells were cultured as previously described for EDJ22 and RW.4 cells^7,8^. Cells were karyotypically normal and cultured for not more than 20 passages and tested bi-monthly for mycoplasma. Undirected differentiation of cells was achieved by depletion of LIF (leukemia inhibitory factor) and beta-mercaptoethanol for at least 4 days as described earlier^7,8^.

Human medulloblastoma cells (Daoy HTB-186, obtained from ATCC) and murine glioblastoma cells (GL261, obtained from ATCC) were cultured in DMEM containing 15% fetal bovine serum.

Neural stem cells were isolated from the dentate gyrus of P0-P2 newborn mice and cultured or differentiated as previously described^7,8^.

Wild-type murine intestinal stem cells were isolated from the intestine of P0-P2 newborn mice and cultured according to Intestinal Epithelial Organoid Culture protocol provided by manufacturer (Stem Cell Technology). Organoids were grown for 7–10 days prior to the experiments.

Wild-type murine hematopoietic stem cells were isolated from the bone marrow of 4–6-week-old mice according to isolation protocol provided by the manufacturer (Miltenyi) and showed 92.97% CD117+ cells (Supplemental Fig. S6C).

Human neuroprogenitor cells were cultured at the Human Embryonic Stem Cell core facility of Washington University as previously described^8^.

### Animal models

Mouse strain C57BL/6 was used for all in vivo studies. Adult 6–8-week-old males were utilized for tissue harvest/sectioning. P0-P2 mouse pups were sacrificed by rapid decapitation prior to dissection for neural and intestinal stem cell isolation. All animal procedures were approved by the Animal Studies Committee at Washington University Medical Center.

### Antibodies

A list of antibodies used in this study is provided in Supplemental Table 2.

### X-ray irradiation and microirradiation

Cells were irradiated with 160 keV X-rays with indicated doses at a dose rate of 1.7 Gy/min in an RS-2000 Biological Research Irradiator (Rad-Source). For microirradiation co-plated embryonic and differentiated cells were cultured for 2 days in 70 μM BrdU and irradiated with a 405 nm and 633 nm laser using a LSM 510 Confocal Microscope (Zeiss, Plan-APOCHROMAT ×63/1.4 oil objective) as previously described^7,8^. ZEN software was used to select cells and target irradiation. Samples were micro-irradiated for 20 min and fixation of cells for immunofluorescence staining was performed 5 min after irradiation.

### RNA interference

Twenty-four hours after plating, cells were transfected using RNAiMAX Lipofectamine (Invitrogen) according to the manufacturer’s instructions. Fifty nM siRNA PP2A (Dharmacon, L-040657-00-0005) was used and incubated 36–48 h before irradiation. According to manufacturer, siRNA products consist of pools of four target-specific siRNAs designed to specifically knockdown gene expression. As control, cells were transfected with the same concentration of non-targeted control siRNA (Dharmacon, D-001810-01-05). Organoids were transfected using Invivofectamin 3.0 (Life Technology Corp., IVF3001).

### Inhibitors

PP2A inhibitor Calyculin A (Tocris, 1336) was dissolved in DMSO and the cells incubated with 0.8 nM inhibitor 2 h before irradiation. Two hours post irradiation, the media containing Calyculin A was removed and fresh media was added. Calyculin A binding affinity to PP2A has been tested using PP2A Immunoprecipitation Phosphatase kit (Milllipore, 2459553) according to the manufacturer’s protocol.

ATM inhibitor (Sigma Aldrich, KU-55933) was dissolved in DMSO and the cells incubated with 10 μM inhibitor for 1 h prior irradiation.

Akt inhibitor (Selleckchem, MK2206) was dissolved in DMSO and the cells incubated with 10 μM inhibitor for 24 h prior irradiation.

### Immunoblot

Cell lysates were obtained by using NP40 buffer (Thermo Scientific, FNN0021) containing protease and phosphatase inhibitor cocktail (Thermo Scientific, 78444) and 1 mM PMSF (Sigma, 78830) according to the manufacturer’s protocol. Protein concentration determined using the BCA protein assay (Thermo Scientific, 23228). Twenty μg of lysate were loaded on a 4–15% tris glycine PAA-Gel (BioRad, 5671083) using tris glycine running buffer (Novex, LC2675). Protein size was determined with the Kaleidoscope prestained marker (BioRad, 161-0375) or HiMark pre-stained protein standard (Novex, LC5699). Transfer to a PVDF membrane was performed with iBlot transfer system (Invitrogen, IB1001). For blocking, the membrane was incubated in 5% milk or BSA in TBS-T for 30 min. Incubation with primary and secondary peroxidase conjugated antibodies and detection of chemiluminescent signal were performed as previously described^7,8^. Quantification has been performed by using ImageJ software. Every western blot performed in this study has been performed at least twice in at least three different cell cultures.

### Apoptosis assay

Cells were trypsinized at indicated timepoints after irradiation and labeled using the FITC Annexin V Apoptosis Detection Kit I (Biomake, B32115) according to the manufacturer’s instructions. At least 5000 cells were analyzed by flow cytometry (Miltenyi, 2550-ANALYZER10).

### Immunofluorescence staining

Cells were grown on cover slips and fixed for 10 min with 4% formaldehyde in PBS and permeabilized for 5 min in 0.2% Triton X-100 in PBS. After several washes in PBS, samples were incubated at least 20 min with 2% BSA in PBS. Incubation with primary or secondary antibody (Supplemental Table 2) was conducted for 1 h at 37 °C. For antibody staining of microscopy samples standard protocols were used as described earlier^7,8^. Samples were mounted in Vectashield mounting medium with (Vector, H1200) or without DAPI (Vector, H1000).

### Immunohistochemistry

Six- to eight-week-old male C57BL/6 mice were sacrificed and tissues frozen in OCT media. Cryosections of 10 μm thickness were obtained from the histology core at Washington University. Frozen sections were thawed in cold PBS, fixed for 20 min in 4% formaldehyde in PBS and permeabilized in 0.2% Triton-X-100 in PBS for 15 min. After several short PBS washes, sections were blocked in 2% BSA in PBS for 1 h. Staining with primary antibodies and secondary antibody incubation was carried out as previously described^7,8^. Cells were stained with well-characterized stem cell-associated markers specific for each tissue, SOX2 for brain, SSEA1/ LGR5 for intestine, and Oct4/ PLZF for testis, respectively.

### Microscopy and image processing

Imaging was performed using a Zeiss Axioplan 2 microscope with ×20, ×63, or ×100 objectives (Plan-NEOFLUAR ×20/0.5, Plan-APOCHROMAT ×63/1.4 Oil, Plan-NEOFLUAR ×100/1.3 Oil) and Meta Systems ISIS imaging software. ImageJ was used to process micrographs, which included cropping of images and minimal adjustment of signal intensity. All images of any experiment were processed in the same way.

### Neutral comet assay

At indicated timepoints after irradiation, cells were trypsinized, centrifuged for 5 min at 300×*g* and resuspended in media to a final concentration of 0.5 × 10^6^ cells per ml.A volume of 5 μl of cell suspension was resuspended in 50 μl pre-warmed (37 °C) 1% agarose (Sigma, A9414), distributed on a glass slide (CometSlide, Trevigen, 4250-200-03) and incubated in cold lysis solution (Trevigen, 4250-050-01) at 4 °C for 1 h. After collection of all samples, electrophoresis and staining with SybrGreen (Life technologies, S7563) and scoring was conducted as previously reported^7,8^.

### Clonogenic assay

Murine embryonic stem cells were treated then irradiated with indicated doses and incubated 6 h after irradiation to allow for DNA repair to occur. Cells were then trypsinized and counted using a Vi-Cell cell counter. The same number of cells for control and siRNA-treated cells were plated and grown for 7 days. Fixation was performed by PBS wash and incubation in cold methanol on ice for 10 min. For staining cells were incubated for 10 min with crystal violet (Sigma, C3886) at RT. Cells were washed twice with water, dried, and counted. Plating efficiency was 15%.

### Cytogenetic analysis

Twenty-four hours after treatment, cells were irradiated at the dose of 2 Gy, which still allow cells to enter mitosis after irradiation. Five hours after irradiation, cells were treated with 100 ng/ml colcemid (Gibco, 15212012) for additional 1 h and 45 min. Mitotic shake-off in cold trypsin was performed, harvested cells were centrifuged for 8 min at 250 × *g* and treated with hypotonic buffer (0.56% KCl) for 8 min. Samples were fixed in acetic acid/methanol (1:3) for at least 45 min and fluorescence-in-situ-hybridization of telomeres, staining and imaging of chromosomes preparation was performed as previously described^7,8^. At least 40 chromosome plates were scored.

### Gene expression profiling

Cells were treated with 10 Gy IR and collected at 15 min and 4 h post IR. Unirradiated cells were used as controls. Total RNA was isolated from cells using RNeasy Kit (QIAGEN, 74104) following manufacturer’s protocol. Integrity of total RNA was determined by Nanodrop ND-1000 analysis. Cyanine-3 (Cy3)-labeled cRNA was prepared from 0.5 μg RNA using the One-Color Low RNA Input Linear Amplification PLUS kit (Agilent, 5188-5339) according to the manufacturer’s instructions, followed by RNAeasy column purification (QIAGEN, 79656). Dye incorporation and cRNA yield were checked with the NanoDrop ND-1000 Spectrophotometer. One microarray was performed for each sample, with no pooling. Samples were hybridized to ArrayStar GPL15692 genechip platform (Arraystar). Microarrays were scanned immediately after washing on a DNA Microarray Scanner (Agilent, G2505B) using one color scan setting for 1 × 44k array slides (scan area 61 × 21.6 mm, scan resolution 10 μm, Dye channel set to Green and Green PMT set to 100%).

### Real-time quantitative RT-PCR

qPCR was conducted to confirm the results for gene expression in samples from all the samples as mentioned earlier using RT2 First strand Synthesis Kit for cDNA synthesis, and amplified using RT2 qPCR Master Mixes in a CFX96 Real-Time PCR (Bio-Rad). We used two sets of primers for qPCR. Forward primer1 was ATG GAC GAG AAG TTG TTC ACC AGG (5′-3′) and reverse primer1 was TTA CAG GAA GTA AGT CTG GGG TAC. Forward primer2 was CCT CTT GTC ATC AAC AGC CGT G and reverse primer2 was GCA GGA AGA ACC CAC AAA GTG. β-actin (ACTB) mRNA was used as an internal reference transcript, with forward primer GCG GGA AAT CGT GCG TGA CAT T and reverse primer GAT GGA GTT GAA GGT AGT TTC GTG.

Gene expression linked to apoptotic pathways has been analyzed by RT² Profiler™ PCR Array Mouse Apoptosis (Qiagen, 330231), according to the manufacturer’s protocol.

### Statistical analysis

Statistical analysis was performed using the two-sided Student’s *t*-test, except for comet assay which has been analyzed using ANOVA. *p*-values of *p* < 0.05 were considered statistically significant and *p* < 0.01 highly statistically significant. Error bars represent the standard deviation of the mean.

Cell culture, neutral comet assay, immunoblot analysis, immunocytochemistry, and immunohistochemistry on adult WT mouse tissue were performed using standard protocols.

Procedures for all experiments including animals were approved by the Animal Studies Committee at Washington University Medical Center.

## Electronic supplementary material

Supplemental Figures and Tables

## References

[1] Crawford JR, MacDonald TJ, Packer RJ (2007). Medulloblastoma in childhood: new biological advances. Lancet Neurol..

[2] Hellstrom NA, Bjork-Eriksson T, Blomgren K, Kuhn HG (2009). Differential recovery of neural stem cells in the subventricular zone and dentate gyrus after ionizing radiation. Stem Cells.

[3] Gondi V, Tome WA, Mehta MP (2010). Why avoid the hippocampus? A comprehensive review. Radiother. Oncol..

[4] Thotala DK, Hallahan DE, Yazlovitskaya EM (2008). Inhibition of glycogen synthase kinase 3 beta attenuates neurocognitive dysfunction resulting from cranial irradiation. Cancer Res..

[5] Thotala DK, Geng L, Dickey AK, Hallahan DE, Yazlovitskaya EM (2010). A new class of molecular targeted radioprotectors: GSK-3beta inhibitors. Int J. Radiat. Oncol. Biol. Phys..

[6] Bartova E (2011). Recruitment of Oct4 protein to UV-damaged chromatin in embryonic stem cells. PLoS ONE.

[7] Jacobs KM (2016). Unique epigenetic influence of H2AX phosphorylation and H3K56 acetylation on normal stem cell radioresponses. Mol. Biol. Cell.

[8] Meyer B (2016). Histone H3 lysine 9 acetylation obstructs ATM activation and promotes iIonizing radiation sensitivity in normal stem cells. Stem Cell Rep..

[9] Neviani P (2013). PP2A-activating drugs selectively eradicate TKI-resistant chronic myeloid leukemic stem cells. J. Clin. Invest.

[10] Tung HY, Pelech S, Fisher MJ, Pogson CI, Cohen P (1985). The protein phosphatases involved in cellular regulation. Influence of polyamines on the activities of protein phosphatase-1 and protein phosphatase-2A. Eur. J. Biochem..

[11] Tung HY, Alemany S, Cohen P (1985). The protein phosphatases involved in cellular regulation. 2. Purification, subunit structure and properties of protein phosphatases-2A0, 2A1, and 2A2 from rabbit skeletal muscle. Eur. J. Biochem..

[12] Chowdhury D (2005). gamma-H2AX dephosphorylation by protein phosphatase 2A facilitates DNA double-strand break repair. Mol. Cell.

[13] Kuo YC (2008). Regulation of phosphorylation of Thr-308 of Akt, cell proliferation, and survival by the B55alpha regulatory subunit targeting of the protein phosphatase 2A holoenzyme to Akt. J. Biol. Chem..

[14] Chang KE (2015). The protein phosphatase 2A inhibitor LB100 sensitizes ovarian carcinoma cells to cisplatin-mediated cytotoxicity. Mol. Cancer Ther..

[15] Gordon IK (2015). Protein phosphatase 2A inhibition with LB100 enhances radiation-induced mitotic catastrophe and tumor growth delay in glioblastoma. Mol. Cancer Ther..

[16] Lv P (2014). Inhibition of protein phosphatase 2A with a small molecule LB100 radiosensitizes nasopharyngeal carcinoma xenografts by inducing mitotic catastrophe and blocking DNA damage repair. Oncotarget.

[17] Wei D (2013). Inhibition of protein phosphatase 2A radiosensitizes pancreatic cancers by modulating CDC25C/CDK1 and homologous recombination repair. Clin. Cancer Res.

[18] Grech G (2016). Deregulation of the protein phosphatase 2A, PP2A in cancer: complexity and therapeutic options. Tumour Biol..

[19] Goodarzi AA (2004). Autophosphorylation of ataxia-telangiectasia mutated is regulated by protein phosphatase 2A. EMBO J..

[20] Dumitru R (2012). Human embryonic stem cells have constitutively active Bax at the Golgi and are primed to undergo rapid apoptosis. Mol. Cell.

[21] Barker N (2007). Identification of stem cells in small intestine and colon by marker gene Lgr5. Nature.

[22] Riehl TE, Santhanam S, Foster L, Ciorba M, Stenson WF (2015). CD44 and TLR4 mediate hyaluronic acid regulation of Lgr5+ stem cell proliferation, crypt fission, and intestinal growth in postnatal and adult mice. Am. J. Physiol. Gastrointest. Liver Physiol..

[23] Harmala-Brasken AS (2003). Type-2A protein phosphatase activity is required to maintain death receptor responsiveness. Oncogene.

[24] Gendron S, Couture J, Aoudjit F (2003). Integrin alpha2beta1 inhibits Fas-mediated apoptosis in T lymphocytes by protein phosphatase 2A-dependent activation of the MAPK/ERK pathway. J. Biol. Chem..

[25] Li X, Nan A, Xiao Y, Chen Y, Lai Y (2015). PP2A-B56 complex is involved in dephosphorylation of gamma-H2AX in the repair process of CPT-induced DNA double-strand breaks. Toxicology.

[26] Yang ZZ (2004). Physiological functions of protein kinase B/Akt. Biochem. Soc. Trans..

[27] Dummler B, Hemmings BA (2007). Physiological roles of PKB/Akt isoforms in development and disease. Biochem. Soc. Trans..

[28] Rivera-Gonzalez GC (2016). Skin adipocyte stem cell self-renewal is regulated by a PDGFA/AKT-signaling axis. Cell Stem Cell.

[29] Fresno Vara JA (2004). PI3K/Akt signalling pathway and cancer. Cancer Treat. Rev..

[30] Dillon RL, White DE, Muller WJ (2007). The phosphatidyl inositol 3-kinase signaling network: implications for human breast cancer. Oncogene.

[31] Tokunaga E (2008). Deregulation of the Akt pathway in human cancer. Curr. Cancer Drug Targets.

[32] Liao Y, Hung MC (2004). A new role of protein phosphatase 2a in adenoviral E1A protein-mediated sensitization to anticancer drug-induced apoptosis in human breast cancer cells. Cancer Res.

[33] Ugi S (2004). Protein phosphatase 2A negatively regulates insulin’s metabolic signaling pathway by inhibiting Akt (protein kinase B) activity in 3T3-L1 adipocytes. Mol. Cell. Biol..

[34] Trotman LC (2006). Identification of a tumour suppressor network opposing nuclear Akt function. Nature.

[35] Hong CS (2015). LB100, a small molecule inhibitor of PP2A with potent chemo- and radio-sensitizing potential. Cancer Biol. Ther..

[36] Ruvolo PP, Clark W, Mumby M, Gao F, May WS (2002). A functional role for the B56 alpha-subunit of protein phosphatase 2A in ceramide-mediated regulation of Bcl2 phosphorylation status and function. J. Biol. Chem..

[37] Chiang CW (2003). Protein phosphatase 2A dephosphorylation of phosphoserine 112 plays the gatekeeper role for BAD-mediated apoptosis. Mol. Cell. Biol..

[38] Shouse GP, Cai X, Liu X (2008). Serine 15 phosphorylation of p53 directs its interaction with B56gamma and the tumor suppressor activity of B56gamma-specific protein phosphatase 2A. Mol. Cell. Biol..

[39] Shouse GP, Nobumori Y, Panowicz MJ, Liu X (2011). ATM-mediated phosphorylation activates the tumor-suppressive function of B56gamma-PP2A. Oncogene.

[40] Li HH, Cai X, Shouse GP, Piluso LG, Liu X (2007). A specific PP2A regulatory subunit, B56gamma, mediates DNA damage-induced dephosphorylation of p53 at Thr55. EMBO J..

[41] Jin Z, Wallace L, Harper SQ, Yang J (2010). PP2A:B56{epsilon}, a substrate of caspase-3, regulates p53-dependent and p53-independent apoptosis during development. J. Biol. Chem..

[42] Deng X, Gao F, May WS (2009). Protein phosphatase 2A inactivates Bcl2’s antiapoptotic function by dephosphorylation and up-regulation of Bcl2-p53 binding. Blood.

[43] Rincon R (2015). PP2A inhibition determines poor outcome and doxorubicin resistance in early breast cancer and its activation shows promising therapeutic effects. Oncotarget.

[44] Arrouss I (2013). Specific targeting of caspase-9/PP2A interaction as potential new anti-cancer therapy. PLoS ONE.

[45] Janzen C, Sen S, Cuevas J, Reddy ST, Chaudhuri G (2011). Protein phosphatase 2A promotes endothelial survival via stabilization of translational inhibitor 4E-BP1 following exposure to tumor necrosis factor-alpha. Arterioscler. Thromb. Vasc. Biol..

[46] Dadey DY (2016). The ATF6 pathway of the ER stress response contributes to enhanced viability in glioblastoma. Oncotarget.

[47] Zhang B (2010). ER stress induced by ionising radiation in IEC-6 cells. Int J. Radiat. Biol..

[48] Saglar E, Unlu S, Babalioglu I, Gokce SC, Mergen H (2014). Assessment of ER stress and autophagy induced by ionizing radiation in both radiotherapy patients and ex vivo irradiated samples. J. Biochem. Mol. Toxicol..

[49] Chang PY (2016). Mensenchymal stem cells can delay radiation-induced crypt death: impact on intestinal CD44(+) fragments. Cell Tissue Res..

[50] Zhu Y, Huang YF, Kek C, Bulavin DV (2013). Apoptosis differently affects lineage tracing of Lgr5 and Bmi1 intestinal stem cell populations. Cell Stem Cell.

[51] Metcalfe C, Kljavin NM, Ybarra R, de Sauvage FJ (2014). Lgr5+ stem cells are indispensable for radiation-induced intestinal regeneration. Cell Stem Cell.

[52] Sato T (2009). Single Lgr5 stem cells build crypt-villus structures in vitro without a mesenchymal niche. Nature.

