# Supplementary material for: Transient PP2A inhibition alleviates normal tissue stem cell susceptibility to cell death during radiotherapy
Source: Cell Death Dis. 2018 Apr 30;9(5):492. doi: 10.1038/s41419-018-0559-0 (PMC5924762; doi:10.1038/s41419-018-0559-0)
Supplement: Supplementary file 1 — Supplemental Figures and Tables [file 41419_2018_559_MOESM1_ESM.pdf]

Supplemental_Fig_S1

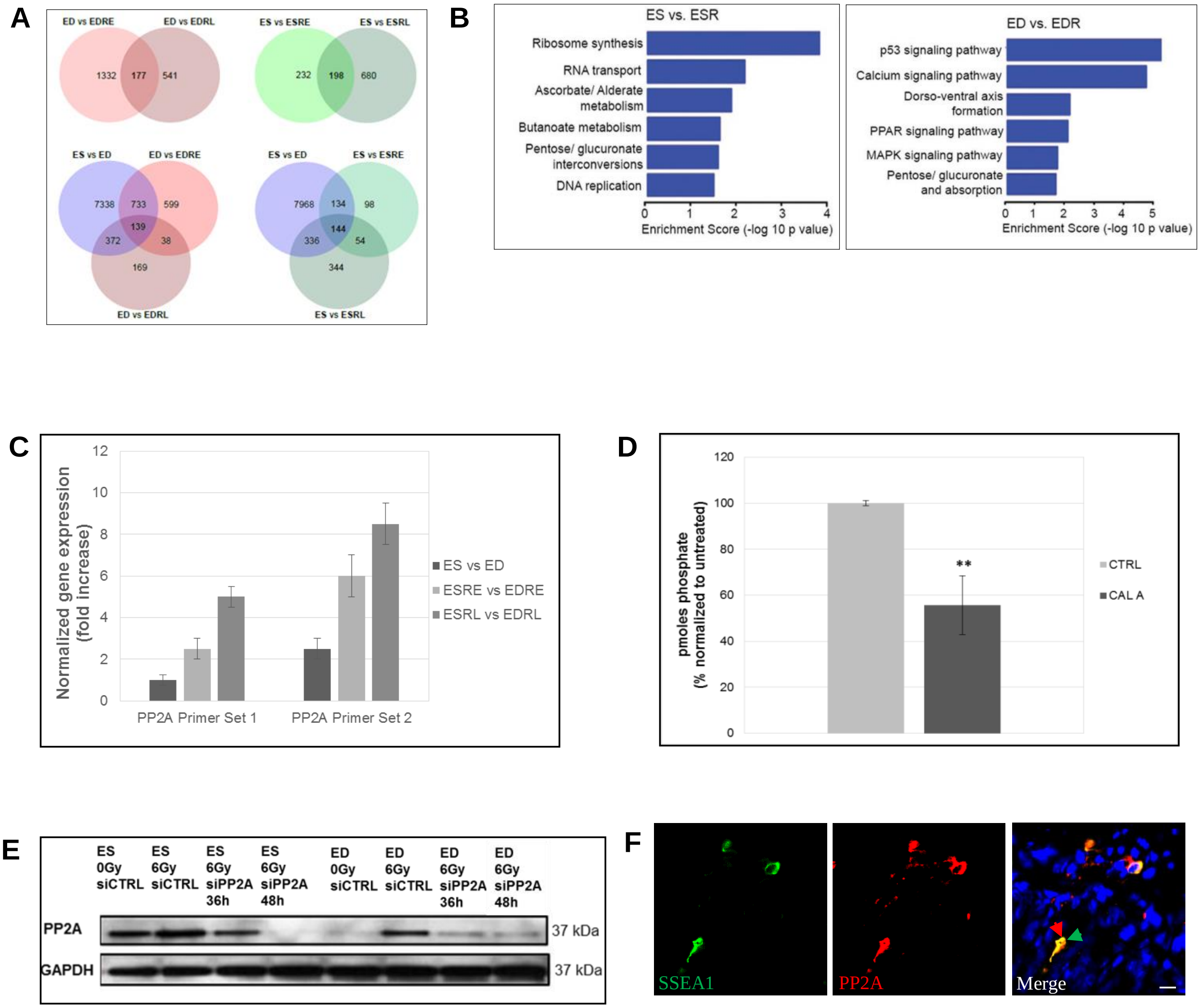

Supplemental_Figure_1S, related to Fig. 1

(A) Shown are Venn diagrams of the number of genes altered upon irradiation at 15 min (RE) and 4 h (RL) and differentiation of ES and ED cells and common intersections.

(B) Shown are pathway enrichment scores of gene expressions altered after IR (early and late time points combined) in ES (left panel) and ED (right panel).

(C) PP2A expression was quantified using qRT-PCR in ES and ED cells 15 min (RE, radiation early) or 4 h (RL, radiation late) after 6Gy IR or without IR. The data were normalized to the expression levels in ED cells.

(D) ES were left untreated or treated with Calyculin A (Cal A) and tested for PP2A immunoprecipitation.

(E) PP2A and GAPDH were detected in lysates of ES and ED cells after siPP2A treatment using immunoblot.

(F) Tissue sections obtained from intestines of WT C57BL/6 mice were stained with SSEA1, PP2A and DNA labeled with DAPI.

Scale bars = 10 μm. Error bars = SD. ** = $p<0.01$

Supplemental_Fig_S2(A)

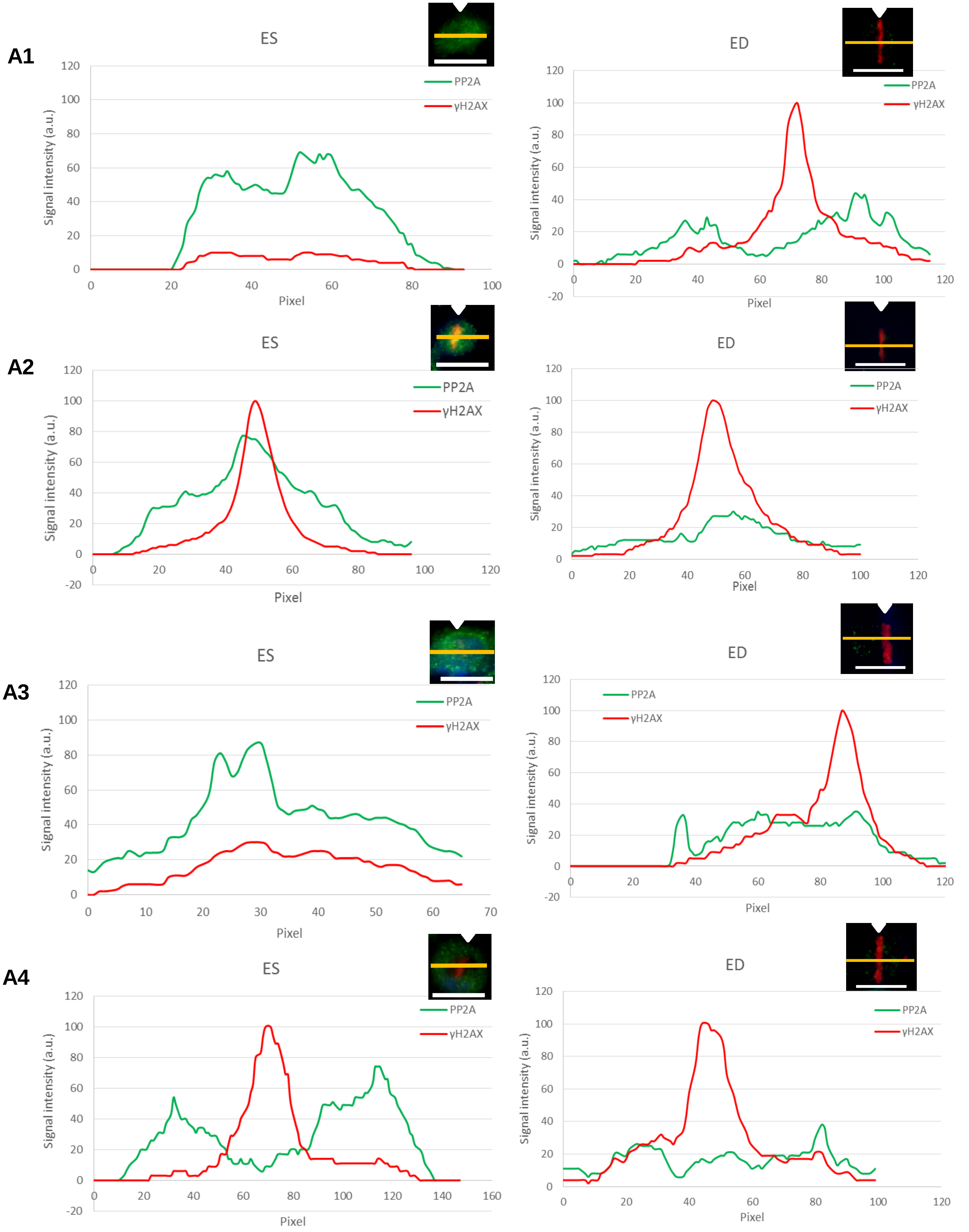

Supplemental_Figure_S2(A), related to Fig. 2

ES cells were co-plated with isogenic non-stem ED cells, microirradiated and fixed immediately after irradiation. PP2A and γH2AX were detected along with Sox2. Cells were subnuclear micro-irradiated in a line across the ES and ED cells in same region of interest (ROI) crossing through both cell types (arrow indicates laser ROI). Quantification of fluorescence intensities along the yellow line is depicted in the corresponding graph.

At least 100 cells have been scored for each treatment. Graphs represent the general pattern observed.

(A1) Untreated ED and ES

(A2) ED and ES cells treated with Calyculin A

(A3) ED and ES cells treated with siCtrl

(A4) ED and ES cells treated with siPP2A

Scale bars = 10 μm.

Supplemental_Fig_S2(B)

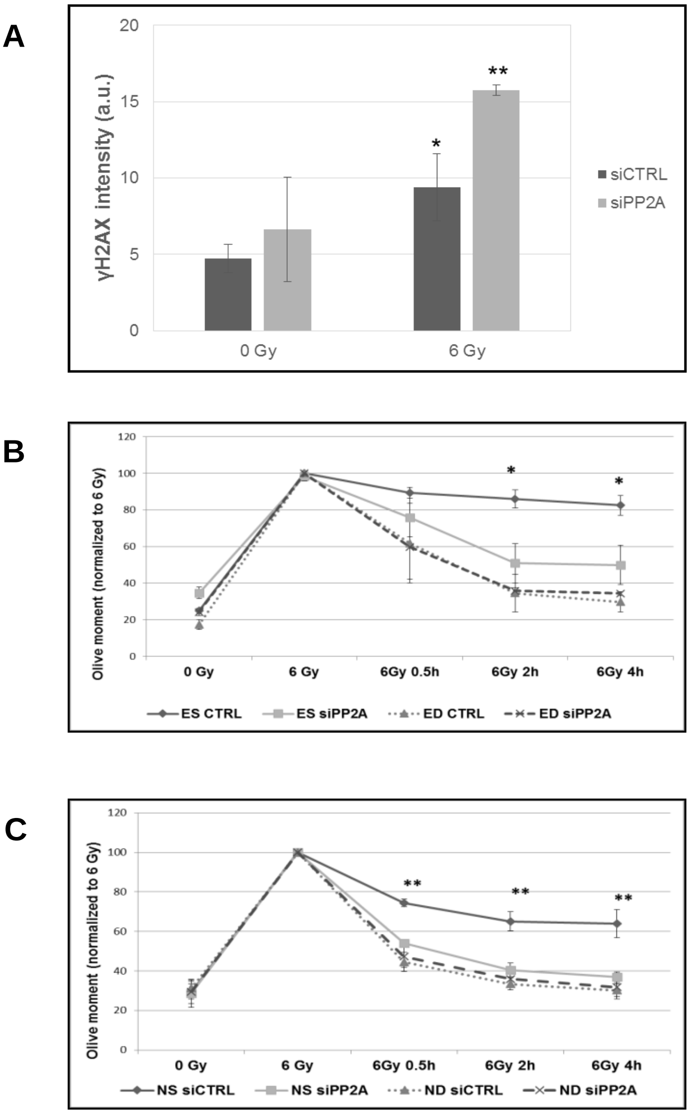

Supplemental_Figure_S2(B), related to Fig. 2

(A) ES cells were treated with siCtrl or PP2A siRNA and γH2AX was analysed by flow cytometry at 0 Gy and 30 minutes after 6 Gy irradiation

(B) ES and ED cells were treated with siCtrl or PP2A siRNA and analyzed by comet assay. Values were normalized to 6Gy time point. 3 independent experiments performed.

(C) NS and ND cells treated with siCtrl or PP2A siRNA and analyzed by comet assay. Values were normalized to 6Gy time point. 3 independent experiments performed.

Supplemental_Fig_S3

A

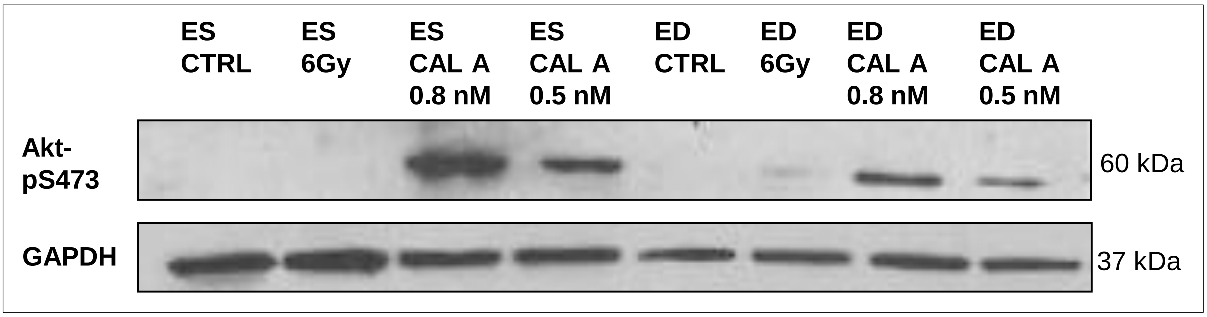

Supplemental_Figure_S3, related to Fig. 3

ES and ED cells were left untreated or treated with Calyculin A (Cal A)

(A) Akt-pS473 and GAPDH were detected in lysates of ES and ED using immunoblot 4 hours after irradiation

A

Treatment	6 Gy	0 Gy CAL A	6 Gy CAL A	0 Gy iAKT	6 Gy iAKT	0 Gy iATM	6 Gy iATM	0 Gy CAL A + iAKT	6 Gy CAL A + iAKT	0 Gy CAL A + iATM	6 Gy CAL A + iATM	0 Gy CAL A + iAKT+iATM	6 Gy CAL A + iAKT +iATM
0 Gy	1.4E-05	1.9E-04	3.0E-05	9.3E-05	5.5E-07	1.5E-03	5.7E-06	7.1E-06	1.2E-06	4.2E-02	8.4E-05	7.9E-05	5.9E-07
6 Gy		4.3E-07	1.5E-04	1.0E+00	1.2E-06	1.5E-05	1.1E-03	8.0E-05	4.3E-06	5.9E-07	1.9E-02	1.8E-01	1.0E-06
0 Gy CAL A			2.7E-07	1.1E-05	1.1E-07	7.0E-06	1.3E-07	1.6E-06	7.2E-08	3.1E-05	1.6E-05	6.8E-06	4.1E-08
6 Gy CAL A				8.1E-03	4.8E-07	3.9E-05	3.4E-06	2.5E-05	4.5E-07	1.3E-07	1.6E-03	1.5E-02	1.4E-07
0 Gy iAKT					8.1E-06	2.7E-04	3.4E-02	2.6E-04	1.1E-04	3.2E-05	3.6E-02	3.3E-01	2.6E-05
6 Gy iAKT						4.0E-07	1.3E-06	1.2E-04	1.4E-05	1.3E-07	3.6E-05	4.3E-06	8.4E-05
0 Gy iATM							3.4E-06	9.4E-06	6.0E-07	5.0E-05	2.0E-04	2.3E-04	2.6E-07
6 Gy iATM								1.6E-04	4.9E-06	7.9E-08	2.2E-01	5.5E-03	8.8E-07
0 Gy CAL A + iAKT									3.3E-01	2.9E-06	1.9E-03	1.3E-04	5.4E-03
6 Gy CAL A + iAKT										6.0E-08	1.3E-03	4.0E-05	1.2E-04
0 Gy CAL A + iATM											4.0E-05	2.1E-05	3.0E-08
6 Gy CAL A + iATM												1.3E-02	2.0E-04
0 Gy CAL A + iAKT+iATM													1.1E-05
6 Gy CAL A + iAKT +iATM													---

Treatment	6 Gy	0 Gy CAL A	6 Gy CAL A	0 Gy iAKT	6 Gy iAKT	0 Gy iATM	6 Gy iATM	0 Gy siCTRL + iAKT	6 Gy siCTRL + iAKT	0 Gy siCTRL + iATM	6 Gy siCTRL + iATM	0 Gy CAL A + iAKT+iATM	6 Gy siCTRL + iAKT +iATM	0 Gy siPP2A + iAKT	6 Gy siPP2A + iAKT	0 Gy siPP2A + iATM	6 Gy siPP2A + iATM	0 GysiPP2A + iAKT+iATM	6 Gy siPP2A + iAKT +iATM
0 Gy	1.4E-05	1.9E-04	3.0E-05	9.3E-05	5.5E-07	1.5E-03	5.7E-06	1.9E-04	3.0E-07	2.9E-01	7.4E-04	2.4E-04	1.9E-05	8.7E-04	9.2E-05	2.6E-04	3.8E-02	1.5E-03	2.6E-04
6 Gy		4.3E-07	1.5E-04	1.0E+00	1.2E-06	1.5E-05	1.1E-03	7.0E-01	2.2E-07	1.1E-05	2.5E-01	7.0E-01	1.1E-04	4.5E-04	2.1E-03	2.0E-07	2.7E-05	7.4E-04	1.2E-02
0 Gy CAL A			2.7E-07	1.1E-05	1.1E-07	7.0E-06	1.3E-07	2.8E-05	1.2E-08	3.1E-04	9.8E-05	3.3E-05	8.0E-06	2.7E-05	2.8E-05	1.8E-02	7.0E-05	5.5E-05	7.6E-05
6 Gy CAL A				8.1E-03	4.8E-07	3.9E-05	3.4E-06	1.5E-02	8.4E-09	2.2E-05	1.9E-01	3.2E-02	5.9E-05	5.3E-03	6.4E-04	3.3E-08	7.5E-05	8.1E-03	2.8E-03
0 Gy iAKT					8.1E-06	2.7E-04	3.4E-02	7.5E-01	1.2E-05	7.3E-05	3.0E-01	7.5E-01	1.7E-04	1.9E-03	3.0E-03	1.1E-05	1.8E-04	2.3E-03	1.5E-02
6 Gy iAKT						4.0E-07	1.3E-06	2.0E-05	2.6E-04	5.0E-07	3.1E-05	1.8E-05	9.4E-02	1.9E-06	5.9E-04	8.7E-08	6.8E-07	3.0E-06	5.1E-04
0 Gy iATM							3.4E-06	6.1E-04	8.3E-08	7.7E-04	3.3E-03	8.6E-04	2.8E-05	1.9E-02	1.8E-04	4.9E-06	1.6E-02	4.0E-02	5.8E-04
6 Gy iATM								1.2E-01	8.9E-08	4.6E-06	3.0E-02	4.8E-02	1.7E-04	1.2E-04	5.1E-03	3.1E-08	9.9E-06	2.2E-04	3.8E-02
0 Gy siCTRL + iAKT									4.0E-05	1.5E-04	2.5E-01	5.7E-01	2.4E-04	3.0E-03	4.5E-03	3.1E-05	3.6E-04	3.4E-03	2.2E-02
6 Gy siCTRL + iAKT										2.7E-07	6.4E-05	3.4E-05	1.3E-01	1.8E-06	3.5E-03	2.1E-09	3.9E-07	3.5E-06	2.2E-03
0 Gy siCTRL + iATM											5.7E-04	1.9E-04	1.8E-05	5.6E-04	8.0E-05	4.6E-04	1.3E-02	9.8E-04	2.3E-04
6 Gy siCTRL + iATM												4.6E-01	2.3E-04	1.8E-02	3.0E-03	1.2E-04	1.5E-03	1.8E-02	1.1E-02
0 GysiCTRL + iAKT+iATM													2.0E-04	4.7E-03	3.4E-03	3.7E-05	4.7E-04	5.2E-03	1.5E-02
6 Gy siCTRL + iAKT +iATM														5.0E-05	4.6E-03	8.5E-06	2.4E-05	5.5E-05	2.8E-03
0 Gy siPP2A + iAKT															3.8E-04	3.0E-05	3.4E-03	8.4E-01	1.3E-03
6 Gy siPP2A + iAKT																3.1E-05	1.3E-04	4.1E-04	2.6E-01
0 Gy siPP2A + iATM																	8.1E-05	6.4E-05	8.6E-05
6 Gy siPP2A + iATM																		6.2E-03	4.0E-04
0 GysiPP2A + iAKT+iATM																			1.3E-03
6 Gy siPP2A + iAKT +iATM																			---

Supplemental_Figure_4S, related to Fig. 4

(A) Statistical analysis relative to Fig. 4A1 and 4A2. Student t test of each endpoint versus any other treatment.

Supplemental_Fig_S5

A

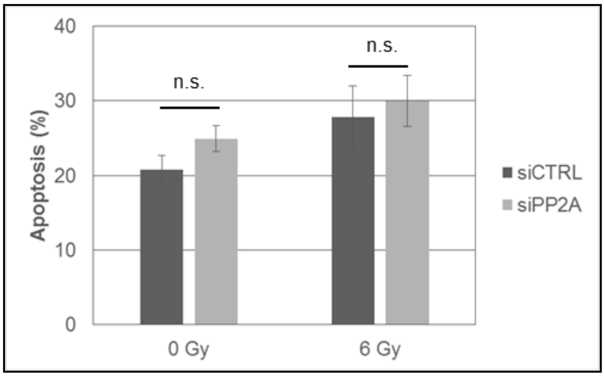

B

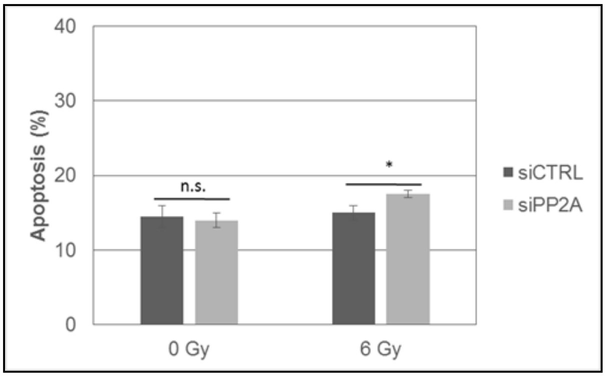

C

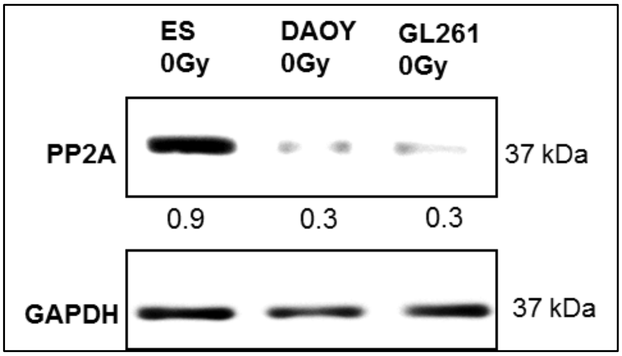

Supplemental_Figure_S5, related to Fig. 5

Cancer cells were left untreated or treated with PP2A inhibitors, irradiated and apoptosis was quantified at 16 h by Annexin V labeling. 3 independent experiments performed. Error bars indicate SD; * $p<0.05$; n.s. not significant

(A) Human medulloblastoma cells (Daoy HTB-186) treated with control (siCtrl) or PP2A siRNA (siPP2A)

(B) Murine glioblastoma cells (GL261) treated with control (siCtrl) or PP2A siRNA (siPP2A)

(C) PP2A and GAPDH analyzed in stem cells (ES), human medulloblastoma cells (DAOY) and murine glioblastoma cells (GL261) by immunoblot.

Supplemental_Fig_S6

A

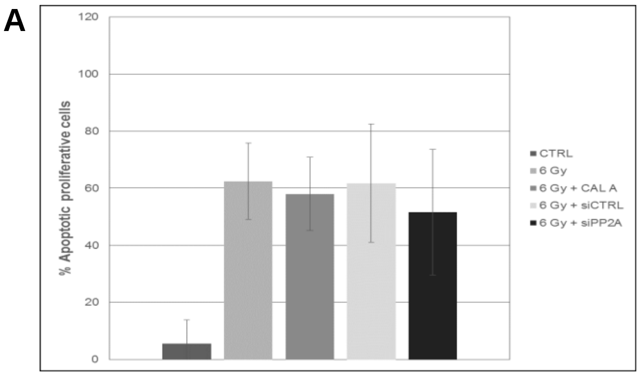

B

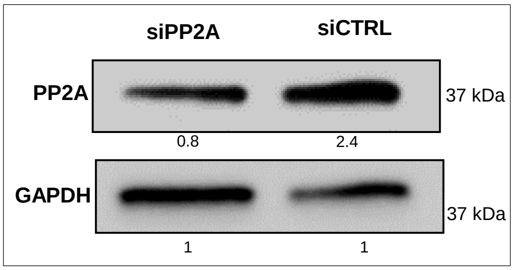

C

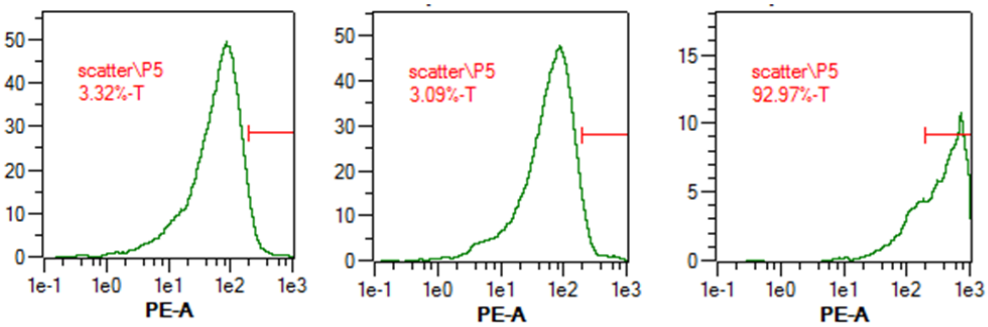

Supplemental_Figure_S6, related to Fig. 6

(A) Murine intestinal organoid proliferative cells were left untreated or irradiated at 6Gy after treatment with Calyculin A (Cal A), control siRNA (siCtrl) or PP2A siRNA (siPP2A). Percentage of cells positive for proliferative cell marker Ki67 and apoptotic marker CC-3 was calculated from total Ki67 positive cells. 3 independent experiments were performed.

(B) PP2A and GAPDH were detected in lysates of intestinal organoids using immunoblot.

(C) Murine hematopoietic stem cells were stained with CD117 antibody and analyzed by flow cytometer. Left panel: unstained control; Middle panel: Isotype; Right panel: CD117 stained.

Supplemental_Table_S1

Lists of stably altered mRNAs from the intersections in Supplemental Fig. S1A			
**Radiation altered mRNAs in undifferentiated stem cells**	**Radiation and Differentiation altered mRNAs in stem cells**	**Radiation altered mRNAs in differentiated non-stem cells**	**Radiation and Differentiation altered mRNAs in non-stem cells**
**ESvsESRE-ESvsESRL**	**ESvsED-ESvsESRE-ESvsESRL**	**EDvsEDRE-EDvsEDRL**	**ESvsED-EDvsEDRE-EDvsEDRL**
Aacs	Aacs	Aanat	Adamts15
Aadac	Ada	Adamts15	Aicda
Acadm	Aldh2	Aicda	Alas2
Ada	Aox3	Alas2	Antxrl
Aldh2	Apoe	Antxrl	Apof
Aox3	Arhgef16	Apof	Atp2b4
Apoe	Atp5c1	Areg	Atp8b3
Arhgef16	BC049807	Atp2b4	B3galt5
Atp5c1	Bet1l	Atp8b3	B9d2
AW551984	Blvrb	B3galt5	Batf
BC049807	Cct8	B9d2	BC057022
Bet1l	Cdc42ep1	Batf	Bfsp2
Blvrb	Cdk2ap2	Bax	Btg2
Ccdc55	Clcnka	BC031353	Casq2
Ccl3	Cnot1	BC051628	Catsper4
Cct8	Csde1	BC057022	Ccdc135
Cdc42ep1	Cstf3	Bfsp2	Ccdc150
Cdk2ap2	Ctf1	Blcap	Ccdc64b
Chchd4	Cybrd1	Bmp15	Ccl21c
Clcnka	Dclre1c	Btg2	Cercam
Cnot1	Ddx28	Bub1	Chrm4
Csde1	Dhrsx	Cacng4	Cma1
Cstf3	Eif3e	Cacng8	Ctxn3
Ctf1	Ep400	Casq2	Cyp4a29
Cybrd1	Eri2	Catsper4	Dhh
D630003M21Rik	Fam18a	Ccdc135	Dleu7
Dclre1c	Gale	Ccdc150	Dll4
Dcun1d4	Gkn2	Ccdc64b	Dusp26
Ddx28	Glrx	Ccl21c	Dusp27
Dennd3	Gm4984	Cercam	Dync1i1
Dhrsx	Gpr107	Chrm4	Eif2c4
Dna2	Grhl1	Ckmt2	Ephx4
Eif3e	H2-Q10	Cma1	Fam101a
Ep400	H3f3a	Cst6	Fam180a

Eri2	Hey2	Ctxn3	Fbxl13
Fam18a	Hps4	Cyp4a29	Fbxo41
Frrs1	Htatip2	Dcxr	Fcrlb
G3bp2	Hunk	Ddit4l	Fgf12
Gale	Inpp5f	Dhh	Fmo4
Gkn2	Ipo8	Dleu7	Fos
Glrx	Klhl5	Dll4	G630016D24Rik
Gm4984	Klra33	Dnajc9	Gal3st1
Gm5148	Loh12cr1	Dusp26	Ggct
Gm525	Lrp10	Dusp27	Gjc3
Gm5797	Lrriq4	Dync1i1	Glb1l3
Gpr107	Mat2b	Eif2c4	Gm11554
Grhl1	Mblac2	Ephx4	Gm13276
Gtf3c4	Med23	Fam101a	Gm1679
H2-Q10	Meox1	Fam180a	Gnat1
H3f3a	Mesdc1	Fbxl13	Got1l1
Hey2	Mllt3	Fbxo41	Gpr18
Hps4	Mtap7d3	Fcrlb	Grhl3
Htatip2	Mustn1	Fgf12	Hebp2
Hunk	Naa16	Fmo4	Hoxc13
Igdcc3	Naaa	Fos	Icos
Inpp5f	Nagk	G630016D24Rik	Ifna5
Ipo8	Nanogpd	Gal3st1	Itgb1bp3
Jph3	Napa	Gcgr	Klk1
Khnyn	Ncor2	Ggct	Krt17
Klhl5	Nedd4l	Gjc3	Krtdap
Klk1b27	Ngfrap1	Glb1l3	Ksr2
Klra1	Nlrp9b	Gm11554	Ldoc1
Klra33	Nr1d2	Gm13276	Lhfpl1
Loh12cr1	Nt5c3	Gm1679	Luzp2
Lrp10	Nt5dc3	Gnat1	Ly6k
Lrriq4	Nub1	Got1l1	Matn1
Map3k9	Oasl2	Gpr18	Mc1r
Mat2b	Ociad2	Grhl3	Mchr1
Mblac2	Panx2	Hebp2	Mtl5
Mcm3	Parp8	Hes6	Myh6
Med23	Patl2	Hoxc13	Nfe2
Meox1	Phactr3	Icos	Nlrp6
Mesdc1	Plekhf1	Ifna5	Nppc
Mfsd11	Plxnb1	Itgb1bp3	Nudt11
Mllt3	Pnpla7	Kap	Nyx
Mos	Ppp2ca	Klk1	Olfr1249
Mrgprg	Pts	Krt17	Olfr1396
Mtap7d3	Rab43	Krtap9-1	Olfr1441
Mttp	Rap1gap	Krtdap	Olfr287
Mustn1	Rapgef1	Ksr2	Olfr371

Naa16	Rbm41	Ldoc1	Olfr713
Naaa	Rnaseh2c	Lhfpl1	Pde1c
Nagk	Rpl12	Luzp2	Pde6b
Nanogpd	Rpl34	Ly6k	Pilra
Nanp	Rpl35a	Matn1	Pklr
Napa	Rpl39l	Mc1r	Pla2g4f
Ncor2	Rpp30	Mcart6	Plcd4
Nedd4l	Rps25	Mchr1	Plin5
Nek8	Sass6	Mtl5	Pln
Ngfrap1	Scarb1	Myh6	Pmaip1
Nlrp9b	Sdc2	Nanos1	Pnliprp2
Nr1d2	Sec61a2	Nfe2	Ppfibp2
Nt5c3	Selenbp2	Nlrp6	Prm2
Nt5dc3	Sema6a	Notch1	Prrxl1
Nub1	Serpinh1	Nppc	Psp
Numbl	Setd7	Nudt11	Ptprcap
Nup43	Sfrs4	Nyx	Ptprt
Oasl2	Skp1a	Olfr1249	Rab40b
Ociad2	Smarcd2	Olfr1396	Rbm3
Olfr1465	Sos1	Olfr1441	Ret
Osgep	Sp3	Olfr287	Rims1
Panx2	Sqrdl	Olfr298	Rnf183
Parp8	St13	Olfr371	Rprm
Patl2	Strap	Olfr697	Ryr2
Pbx2	Synj2	Olfr713	Sema6d
Pcsk9	Tagln2	Pard6g	Shisa7
Pfkfb3	Tbc1d9	Pcdhb22	Shroom3
Phactr3	Tcf25	Pde1c	Slc26a4
Plekhf1	Tctex1d2	Pde6b	Slc26a9
Plxnb1	Thap4	Pilra	Slc37a2
Pnpla7	Tmc5	Pitpnc1	Slc45a1
Pop1	Tmem151b	Pklr	Slc5a11
Ppp2ca	Tmem17	Pla2g4f	Slc6a14
Pts	Tmem219	Plcd4	Smtnl1
Rab43	Tmem43	Plin5	Sostdc1
Rap1gap	Tmem69	Pln	Spink5
Rapgef1	Tmod2	Pmaip1	Stox2
Rbm41	Tnks1bp1	Pnliprp2	Tas2r143
Rnaseh2c	Ttn	Ppfibp2	Tcte3
Rpl12	Ube2i	Prdm10	Tmem44
Rpl19	Usp5	Prm2	Tmem51
Rpl34	Vps28	Prrxl1	Tmhs
Rpl35a	Wnk2	Psg25	Tnfrsf18
Rpl36al	Zbtb5	Psp	Trp53inp1
Rpl39l	Zbtb8os	Ptprcap	Tspan33
Rpp30	Zzef1	Ptprt	Tssk3

Rps25		Rab40b	Ube2j2
Rps27a		Rbm3	Ugt2b37
Rtn4r		Ret	Unc50
Sass6		Rims1	Zfp874
Scarb1		Rltpr	Zpbp2
Sdc2		Rnf183	
Sec61a2		Rprm	
Selenbp2		Ryr2	
Sema6a		Selplg	
Serpinh1		Sema6d	
Setd7		Shisa7	
Sfrs4		Shroom3	
Skp1a		Slc26a4	
Slc16a7		Slc26a9	
Smarcd2		Slc37a2	
Smcr7		Slc45a1	
Sos1		Slc5a11	
Sp3		Slc6a14	
Sp7		Smtnl1	
Sqrdl		Sostdc1	
St13		Spink5	
Stk16		St18	
Strap		Stox2	
Synj2		Strc	
Tagln2		Suox	
Tbc1d9		Tas2r143	
Tcf25		Tcte3	
Tctex1d2		Tmem44	
Teddm1		Tmem51	
Thap4		Tmhs	
Tmc5		Tmod4	
Tmem151b		Tnfrsf18	
Tmem17		Trp53inp1	
Tmem219		Tspan33	
Tmem43		Tssk3	
Tmem69		Ube2j2	
Tmem8		Ugt2b37	
Tmod2		Unc50	
Tnks1bp1		Zfp385a	
Ttn		Zfp874	
Ube2i		Zpbp2	
Usp5			
Utp14b			
Vps28			
Wnk2			
Zbtb5			

Zbtb8os			
Zfp428			
Zzef1			

Supplemental Table 2. List of antibodies used in this study

Protein target	Species	Company	Catalog #o	Assay	Conditions
AMCA-conjugated anti-mouse	horse	Vector	FI-2100	IF, IHC-F	1:100
AMCA-conjugated anti-rabbit	donkey	Vector	FI-1200	IF, IHC-F	1:100
Bax	mouse	Santa Cruz	sc-7480	WB	1:250 in milk
Bcl-2	rabbit	Cell Signaling	2870	WB	1:100 in milk
CD117-PE	mouse	Miltenyi	130-102-542	IF	-
Cleaved Caspase-3	rabbit	Cell Signaling	9664	IHC-F, WB	1:200, 1:500 in milk
FITC-conjugated anti-mouse	horse	Vector	FI-2100	IF, IHC-F	1:100
FITC-conjugated anti-rabbit	donkey	Vector	FI-1200	IF, IHC-F	1:100
GAPDH	mouse	Sigma Aldrich	G8795	WB	1:100 in milk
HRP-conjugated anti-mouse	rabbit	Sigma Aldrich	A9044	WB	1:5000 in milk
HRP-conjugated anti-rabbit	goat	Sigma Aldrich	A0545	WB	1:5000 in milk
Isotype IgG2b-PE	rat	Miltenyi	130-102-663	IF	-
Ki67	mouse	UltraMab	UM800033	IF	1:100
Oct 4	rabbit	Abcam	ab19857	IF, IHC-F, WB	1:200, 1:200, 1:1000 in milk
phospho-AKT	rabbit	Cell Signaling	4060	WB	1:1000 in milk
phospho-ATM (S1981)	mouse	Cell Signaling	4526	IHC-F, WB	1:50, 1:500 in milk
PP2A	mouse	Millipore	05-421	IF, IHC-F, WB	1:200, 1:200, 1:1000 in milk
Sox2	rabbit	Abcam	ab97959	IHC-F, WB	1:100, 1:1000 in milk

Sox2	mouse	Abcam	ab79351	IF, IHC-F	1:200, 1:100
SSEA1	mouse	Abcam	ab16285	IHC-F, WB	1:100, 1:1000 in milk
Texas Red-conjugated anti-mouse	horse	Vector	FI-2100	IF, IHC-F	1:100
Texas Red-conjugated anti-rabbit	donkey	Vector	FI-1200	IF, IHC-F	1:100
γH2AX (S139)	mouse	Millipore	05-636	IHC-F, WB	1:1000, 1:4000 in milk
